# Controllable Growth of Ultrathin P-doped ZnO Nanosheets

**DOI:** 10.1186/s11671-016-1379-8

**Published:** 2016-04-01

**Authors:** Yuankun Zhu, Hengyan Yang, Feng Sun, Xianying Wang

**Affiliations:** School of Materials Science and Technology, University of Shanghai for Science and Technology, Shanghai, 200093 China

**Keywords:** Ultrathin ZnO nanosheets, Phosphor doping, Oxygen, Controllable growth

## Abstract

Ultrathin phosphor (P)-doped ZnO nanosheets with branched nanowires were controllably synthesized, and the effects of oxygen and phosphor doping on the structural and optical properties were systematically studied. The grown ZnO nanosheet exhibits an ultrathin nanoribbon backbone with one-side-aligned nanoteeth. For the growth of ultrathin ZnO nanosheets, both oxygen flow rate and P doping are essential, by which the morphologies and microstructures can be finely tuned. P doping induces strain relaxation to change the growth direction of ZnO nanoribbons, and oxygen flow rate promotes the high supersaturation degree to facilitate the growth of nanoteeth and widens the nanoribbons. The growth of P-doped ZnO in this work provides a new progress towards the rational control of the morphologies for ZnO nanostructures.

## Background

As a direct band gap (3.37 eV) semiconductor with large exciton binding energy (60 meV), ZnO has been extensively studied due to its potential applications in light-emitting devices (LEDs) [1], lasers [2], solar cells [3], supercapacitors [4], and nanogenerators, [5] etc. ZnO nanostructures exhibit the most abundant morphologies, such as nanowires, nanoneedles, nanorods, nanobelts, nanosheets, and nanorings, etc. [6–11]. Diverse morphologies of ZnO make it a promising material in various innovative nanodevices. Typically, to develop nanodevices based on ZnO, complementary doping including n-type and p-type is necessary. However, ZnO nanostructures with stable p-type doping are difficult to reproduce because of its self-compensation and low solubility of dopants [12]. Various elements of N, S, P, As, Cu, and Sb have been considered as dopants to produce p-type ZnO [6, 13–17]. Phosphor (P) doping was reported to be effective in modifying the electrical conducting performances of ZnO nanostructures [12, 17, 18], whereas P doping induces variation of microstructures and optical properties of ZnO. Our previous work showed that P doping in ZnO nanostructures restrained the growth of ZnO along a [0001] direction, resulting in the morphology evolution from nanorods to nanoplatelets [19], and a similar phenomenon was observed in other works [17]. Though many efforts have been performed, controllable growth of P-doped ZnO nanostructures with desired morphologies and properties is still challenging, which requires comprehensive understanding on the growth mechanism of ZnO nanostructures by P doping.

Thin ZnO nanosheets are considered to exhibit unique advantages in applications of nanophotonics, nanosensor arrays, and nanocantilevers, etc. [18, 20–22]. ZnO nanosheets with or without branched structures have been synthesized by various methods, including chemical vapor deposition [9, 17], thermal evaporation [18], and hydrothermal methods [23]. Depending on growth temperatures, precursors, or substrates, ZnO nanosheets with diverse morphologies (i.e., double-sided, one-sided, tapered teeth) could be fabricated. However, ultrathin P-doped ZnO nanosheets were seldom synthesized, and very few reports focused on the effect of carrier gases on the formation of branched nanostructures. Tailoring the morphologies by controlling the carrier gas provides a simple yet effective way in synthesizing ultrathin ZnO nanosheets with branched structures. In this work, we demonstrated that such structural revolution process of ZnO could be tuned by oxygen content in carrier gas, and ultrathin P-doped ZnO nanosheets with branched structures can be reproducibly produced at a proper oxygen flow rate. Meanwhile, the effect of P doping on the morphology evolution was studied in detail, and the growth mechanism of ultrathin P-doped ZnO nanosheets with branched structures was comprehensively investigated.

## Methods

Ultrathin P-doped ZnO nanosheets were grown by using a double-tube chemical vapor transporting and condensation (CVTC) system equipped with a Lindberg blue tube furnace. Prior to growth, the temperature inside the furnace was carefully calibrated by using a K-type thermocouple (TES 1310, Taiwan). Equal amounts of ZnO (Alfa Aesar 99.99 %) and graphite (200 mesh, Alfa Aesar 99.9 %) powders mixed with phosphor pentoxide (P_2_O_5_, Alfa Aesar 99.99 %) nanopowders were loaded into a high-purity alumina boat (Al_2_O_3_ 99.5 %), which was positioned at the center of the tube during growth. An a-plane sapphire substrate coated with a thin Au catalyst layer was used as the substrate. High-purity Ar (99.999 + %) and O_2_ (99.999 + %) with different ratios were used as the carrier (reaction) gases at a constant total flow rate of 110 sccm. The furnace was heated to 1000 °C with a ramping rate of 50 °C/min, and the growth time was 5 min for all samples. All of the ZnO samples were grown under environmental pressure.

The surface morphologies of as-grown ZnO samples were investigated by using a field-emission scanning electron microscope (FE-SEM, FEI, Quanta FEG) and atomic force microscope (AFM, Asylum Research, Cypher). The crystal structures of ZnO nanosheets were characterized by using an X-ray diffractometer (XRD, Brukes, D8 Advance) with Cu *K*α radiation at a wavelength of 0.154 nm. Raman spectra of ZnO nanosheets were examined by using a Raman station 400 F machine (PerkinElmer). Room-temperature photoluminescence (PL) spectra were collected by using an FS920-type spectrometer (Edinburgh) with an exciting wavelength of 325 nm.

## Results and Discussions

### Surface Morphology

**Fig. 1:**
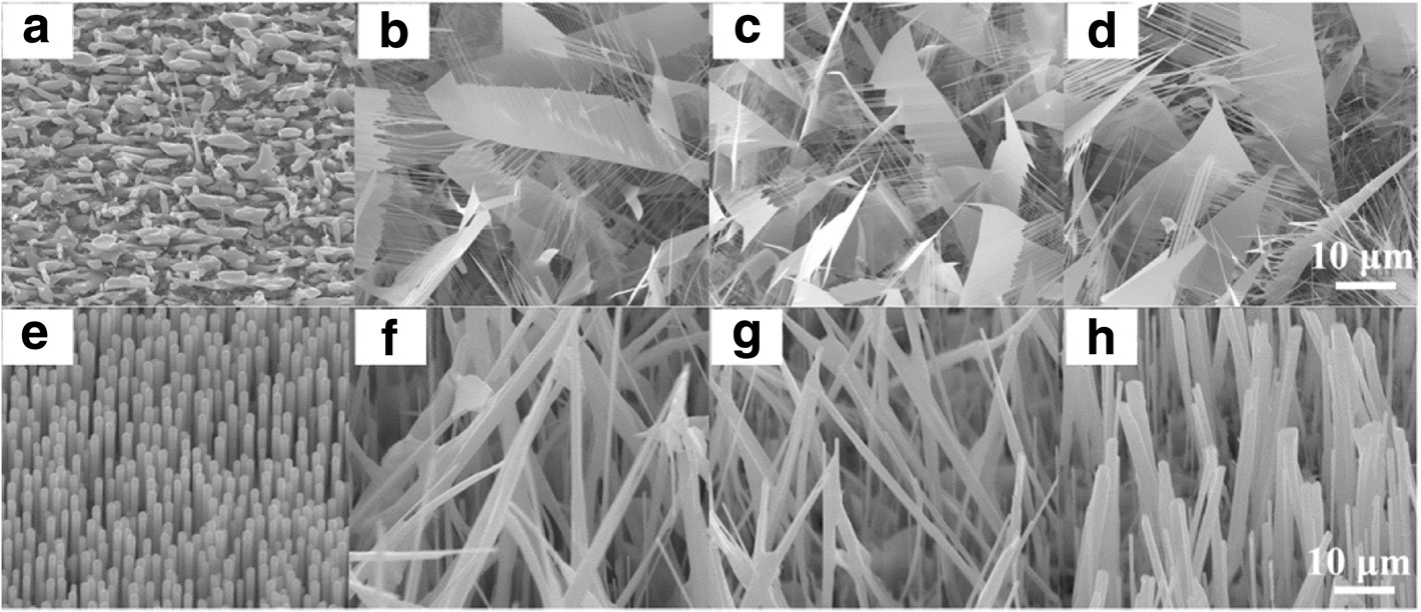
**a**–**d** SEM images of ZnO nanosheets with 2.5 % P doping grown at oxygen flow rates of 0, 10, 20, and 30 sccm, respectively. **e**–**h** SEM images of undoped ZnO nanowires grown at oxygen flow rates of 0, 10, 20, and 30 sccm, respectively

Figure 1a–d shows the typical SEM images of P-doped ZnO nanostructures grown at different oxygen flow rates. Clearly, the morphologies of ZnO nanostructures are changed dramatically with the variation of oxygen flow ratio. Without oxygen flow gas, ZnO nanoparticles are formed at the initial growth stage due to the high Zn vapor concentration; then, with the consumption of Zn vapor, small amounts ZnO nanowires begin to grow on the nanoparticles, resulting in the morphologies shown in Fig. 1a. When oxygen is introduced into the tube, ZnO nanosheets consisting of ribbon-like backbone and parallel nanoteeth are synthesized (Fig. 1b). The nanoteeth are grown epitaxially out of large ribbons with a length of tens of micrometers. When oxygen flow rate increases to 20 sccm, the width of nanoribbons increases, meanwhile an irregular shape is observed, indicating the growth direction changes to the transverse direction in Fig. 1c. The variation of growth direction for ZnO is attributed to the strain relaxation along the transverse direction, which results from P atoms inducing lattice distortion in ZnO. Further increasing oxygen flow rate promotes the continuous growth of nanoribbons along its transverse direction. TEM characterization identifies that the growth of nanoribbons is along the $\left[10\overline{1}0\right]$ direction and enclosed by two large ±$\left[1\overline{2}10\right]$ surfaces, while the growth of nanoteeth is along the [0001] direction of ZnO [24]. To further confirm the effect of oxygen flow gas, ZnO nanostructures without P_2_O_5_ were grown at different oxygen flow rates, and the SEM images of undoped ZnO nanostructures are shown in Fig. 1e–h. Evidently, without P_2_O_5_, only ZnO nanowires are synthesized. With the increase of oxygen flow ratio, the length of ZnO nanowires increases along the *c*-axis, which demonstrates the acceleration effect of oxygen for the growth of undoped ZnO nanostructures. From Fig. 1, it can be concluded that both oxygen flow gas and P_2_O_5_ are decisive to the synthesis of ZnO nanosheets, and oxygen accelerates the growth of P-doped or undoped ZnO nanostructures.

**Fig. 2:**
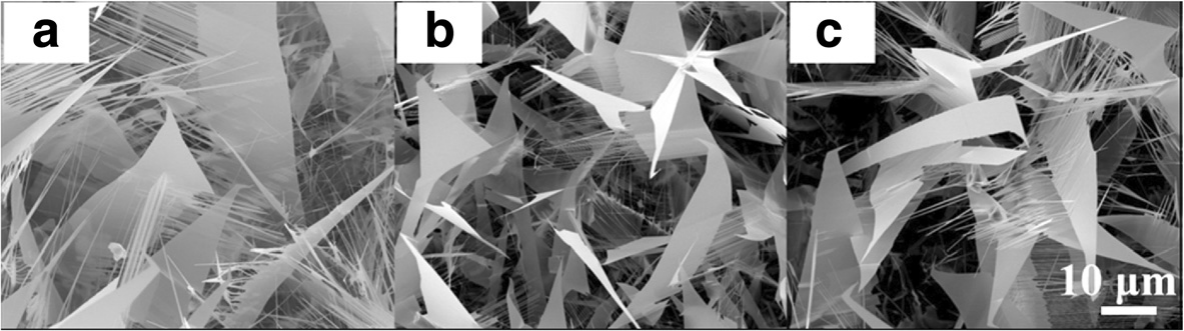
SEM images of ZnO nanostructures grown at a constant oxygen flow rate of 30 sccm with P_2_O_5_ weight ratios of **a** 2.5, **b** 5, and **c** 10 %, respectively

Figure 2a–c shows SEM images of ZnO nanostructures with different P_2_O_5_ weight ratios grown at a constant oxygen flow rate of 30 sccm. With the increase of P_2_O_5_ weight ratio, the surface morphology of ZnO nanosheets does not change significantly, which is probably due to the low solubility of P atoms in ZnO. In addition, it is observed that with the increase of P_2_O_5_ weight ratio, the area size of ZnO nanosheets gradually decreases, which is attributed to the limited capacity of an alumina boat leading to the reduction of ZnO source powders with the increase of P_2_O_5_ nanopowders. From Figs. 1 and 2, it demonstrates that oxygen flow gas and P_2_O_5_ are essential for the synthesis of ZnO nanosheets.

**Fig. 3:**
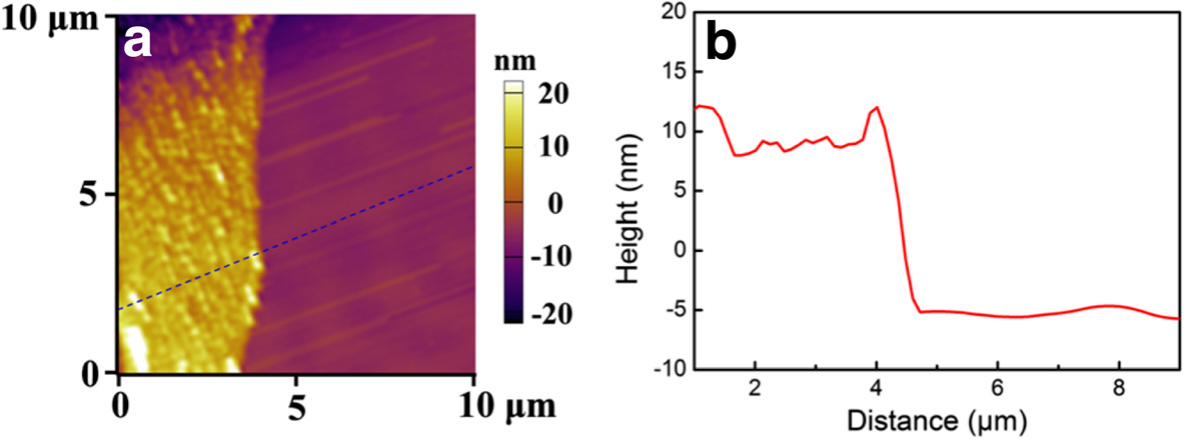
**a** Typical AFM image and **b** corresponding cross-sectional height curve of P-doped ZnO nanosheets

It should be noted that the ZnO nanosheets are almost transparent as observed from the SEM images, indicating the ultrathin feature of ZnO nanosheets. To accurately characterize the thickness of P-doped ZnO nanosheets, AFM is utilized to measure the cross-sectional height of ZnO nanosheets. Figure 3 shows the typical AFM image and the corresponding cross-sectional height curve of ZnO nanosheets. It can be seen that the grown ZnO nanosheet shows a quite small thickness of about 13–15 nm, compared with its length of tens of micrometers. From SEM and AFM observations, it concludes that ultrathin P-doped ZnO nanosheets with branched structures are synthesized in this study.

### Crystal Structure

**Fig. 4:**
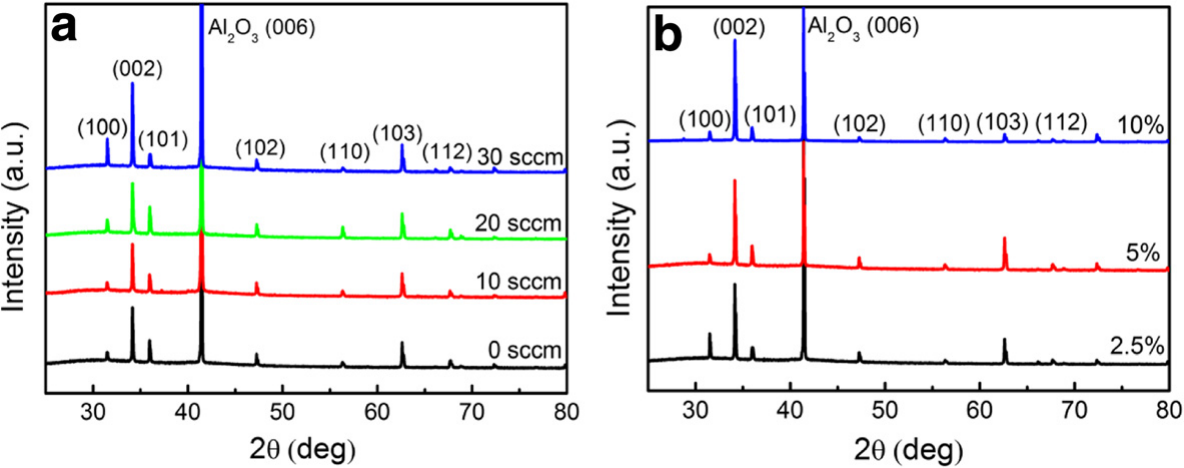
X-ray diffraction pattern of P-doped ZnO nanostructures grown at **a** different oxygen flow rates and **b** different P_2_O_5_ weight ratios

XRD patterns of ZnO nanosheets grown at different oxygen flow rates are shown in Fig. 4a. Strong (002), (101), (100), and (103) peaks as well as several small peaks assigned to wurtzite ZnO are observed for all of the samples, and the P-related diffraction peaks are not observed within the detection limit of XRD detection. With the increase of oxygen flow rate, the full width at half maximum (FWHM) of the dominated (002) peak slightly decreases. Compared with the standard PDF card #36-1451 for pure bulk ZnO, a small shift (0.2~0.3°) towards a smaller angle direction is observed for all of the diffraction peaks, indicating that the *c*-axis lattice constant of ZnO nanosheets is tuned. According to the Bragg diffraction law, the *c*-axis lattice constant of ZnO nanosheets remains at 0.5241 nm with the increase of oxygen flow rate, while pure ZnO material shows a lattice constant of 0.5206 nm (PDF#36-1451). The larger lattice constant of P-doped ZnO nanosheets is due to the larger bond of Zn-P than ZnO by P doping, demonstrating that P atoms completely substitute for O or Zn. With the variation of growth parameters, the asymmetric peak shapes do not change obviously. The relative intensity ratio of the (002) diffraction peak to the other diffraction peaks ((101) or (103)) is gradually strengthened with the increase of oxygen flow rate, indicating that an oxygen-enriched environment is favorable for the growth along the *c*-axis of ZnO, which is consistent with previous SEM characterization. Figure 4b shows the XRD patterns of ZnO nanosheets grown with different P_2_O_5_ weight ratios. Similarly, strong (002) peaks are observed for all of the ZnO nanosheets. No evident variation of the diffraction patterns are observed for ZnO nanosheets with different P_2_O_5_ ratios. The FWHM of the dominated (002) peak are, respectively, 0.1957°, 0.1968°, and 0.1975° for the 2.5, 5, and 10 % P_2_O_5_ samples. It can be seen that with the increase of P_2_O_5_ ratio, the FWHM of ZnO nanosheets gradually increases. It should be pointed out that though P-related peaks are not observed by XRD characterization, the compositions of P-doped ZnO nanosheets have been characterized by X-ray photoelectron spectroscopy (XPS) in our previous study [24]. P-related peaks located at 187.1 eV (P 2s) and 131 eV (P 2p_3/2_) were observed, and it identified that P was negatively charged (P^3−^), confirming that P dopants were essentially incorporated to the ZnO nanosheets and bonded with Zn to occupy O site.

### Raman Spectra

**Fig. 5:**
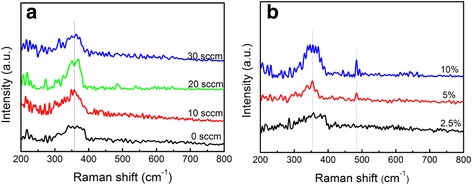
Raman spectra of P-doped ZnO nanostructures grown at **a** different oxygen flow rates and **b** different P_2_O_5_ weight ratios

Raman signals of ZnO are sensitive to its defects and structure. Wurtzite ZnO belongs to the P63mc space group and its crystal symmetry is C6V4; the center of the Brillion optical phonon is two A1, two E1, two E2, and two B1 modes. Normally, A1 (LO) located at 574 cm^−1^ and E2 (high) modes located at 437 cm^−1^ can be observed [25]. Figure 5a shows the Raman spectra of ZnO nanosheets grown at different oxygen flow rates. Only one peak located at 360~370 cm^−1^ is observed for all of the ZnO samples, which clearly does not belong to any vibrating modes of pure ZnO. This vibrating peak is attributed to the local vibrational mode (LVM) induced by P dopants substituting O atoms [26]. With the increase of oxygen flow rate, the relative intensity of LVM decreases due to the suppression effect of more oxygen atoms diffusing into ZnO lattices, which confirms that crystal quality of ZnO nanostructures can be improved in an oxygen-enriched environment.

Figure 5b shows the Raman spectra of ZnO nanosheets grown with different P_2_O_5_ weight ratios. Similarly, a peak located at 360~370 cm^−1^ is observed for all of the ZnO samples. Different from ZnO samples grown at different oxygen flow rates, with the increase of P_2_O_5_ weight ratio, a new peak located at 482 cm^−1^ appears, which is attributed to another LVM of P corporation with ZnO to substitute Zn atoms. According to the model for rough estimation of P-related LVM frequencies $\omega \left(\mathrm{L}\mathrm{V}\mathrm{M}\right)/\omega \left(\mathrm{Z}\mathrm{n}\mathrm{O}\right)=\sqrt{\mu_{\mathrm{ZnO}}/{\mu}_{\mathrm{LVM}}}$, where *ω* is the vibrational frequency and *μ* is the effective mass [25], the calculated values for LVMs of P on substitutional Zn and O sites in ZnO lattice are, respectively, 482 and 341 cm^−1^, which are consistent with the experimentally observed values of 482 and 360–370 cm^−1^, confirming the localization of P in ZnO. This result is in accordance with the XPS investigation in our previous work that P-related peaks located at 187.1 eV (P 2s) and 131 eV (P 2p_3/2_) were observed, demonstrating that P atoms were essentially incorporated to the ZnO nanosheets [24]. Similar LVMs located at 478 and 364 cm^−1^ were observed in P-doped ZnO films by other group [26]. A possible physical mechanism of the LVMs is that P dopants break the translational symmetry of ZnO, thereby relaxing the conservation of wave vector; and they can lead to scattering by phonons in ZnO materials that have wave vectors far from the zone center. LVMs of ZnO films doped with Fe, Sb, Al, Ga, Co, V, and N have also been reported [25].

It has been observed that undoped ZnO showed a predominant A1 (TO) mode at 382 cm^−1^ and a predominant E2 (high) mode at 438 cm^−1^ by Raman characterization in our previous study [27], while in this work no obvious Raman peak assigned to wurtzite ZnO is observed for all of the P-doped ZnO samples. A similar phenomenon has been observed by J. D. Ye et al. that for undoped ZnO, a clear Raman peak for the wurtzite structure was observed while no peak assigned to the wurtzite structure could be identified for P-doped ZnO [26]. The absence of the Raman vibrational peak for the wurtzite structure in P-doped ZnO might de due to the P dopants inducing not only topological disorder but also structural disorder. These disorders result basically in the breakdown of translational symmetry of the periodic lattice, which yields a partial relaxation of the *q* = 0 selection rule for Raman scattering.

### Photoluminescence Spectra

**Fig. 6:**
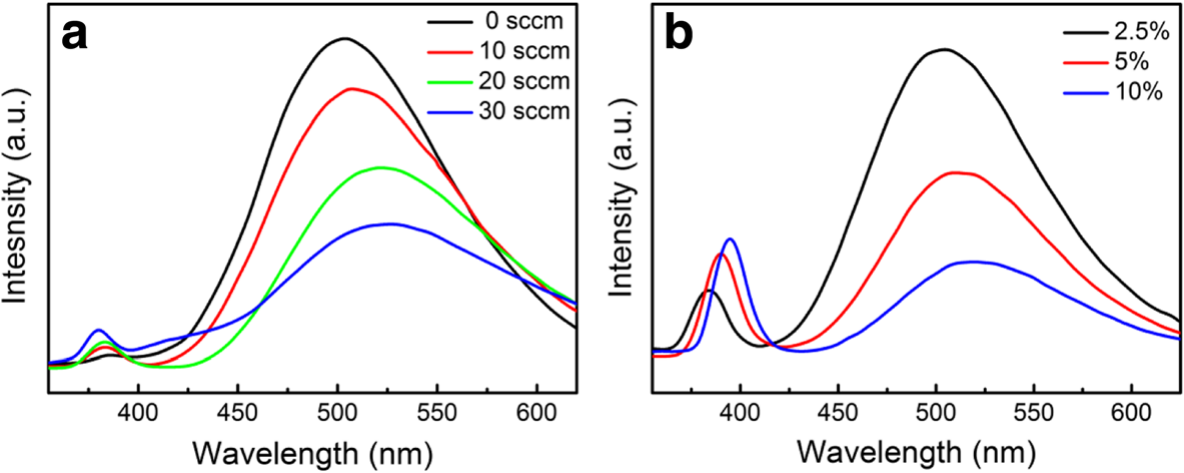
PL spectra of P-doped ZnO nanostructures grown at **a** different oxygen flow rates and **b** different P_2_O_5_ weight ratios

The PL emission of nanomaterials commonly is attributed to three different physical origins: self-trapped excitons, oxygen vacancies, and surface states (defects) [28]. For ZnO, the near-band-edge (NBE) exciton UV emission and defect-related deep-level green emission (DLE) are usually observed. Figure 6a shows the PL spectra of P-doped ZnO nanosheets grown at different oxygen flow rates. The ZnO nanosheets exhibit weak UV emission peaks located at 383 nm and strong green peaks centered at around 501–524 nm. The UV emission band is attributed to a near-band-edge transition of ZnO, namely the recombination of free exciton emission process, and the green band is related to the radial recombination of a photogenerated hole with the electron in a singly ionized oxygen vacancy [29]. Several features can be found from the PL spectra of P-doped ZnO nanosheets. The intensities of defect-related peaks are much stronger than those of the NBE emission for all samples, indicating that many defects such as oxygen vacancies are generated during the growth. The intensity of the UV peak increases and the deep-level emission is suppressed with the increase of oxygen flow rate; the relative intensity ratios of the two peaks are 0.035, 0.08, 0.14, and 0.26, respectively. In addition, it is observed that the UV peaks are blue-shifted with the increase of oxygen flow rate, whereas the green peaks are red-shifted.

It is reasonable that the UV emission shifts with the change of oxygen flow ratio, and similar phenomena have been observed in P-ZnO and SnO_2_ materials [30, 31]. The UV emission belongs to the near-band-edge transition, which is associated with the bandgap. The shift of UV emission is probably due to the variation of bandgap of ZnO via the band tailing effect. The tailing of the band-edge (Urbach tail) into the forbidden gap region (that correlates to the sharpness of absorption edge) depends on the structural disorder, states due to impurities and defects, carrier concentration, and temperature [31]. With the increase of oxygen flow rate, there are fewer defects in ZnO and the crystal quality of ZnO would be improved, which weakens the band tailing effect and changes the tail width; thus, the bandgap of ZnO would increase, which results in the blue shift of UV emission. By low-temperature (10 K) PL characterization, Kwon et al. pointed out that the green emission peaks were mainly originated from native defects and P dopants in undoped and doped ZnO, respectively, and P doping in ZnO would reduce the native deep acceptor states [32]. Besides, there was a trade off between a suppression of native defects and an incorporation of P dopants. In this study, the broad green emission peak observed at room temperature would result from emissions of both native defects and P dopants. Thus, the broad green emission peak shifts by the variation of native defects and/or P dopants. With the increase of oxygen flow rate, more oxygen atoms are introduced into the ZnO, and less oxygen vacancies would be produced; thus, the emission from native defects would be suppressed, leading to the shift of broad green emission peak and the intensity variation.

Figure 6b shows the PL spectra of ZnO nanosheets with different P_2_O_5_ weight ratios. It is observed that with the increase of P_2_O_5_ weight ratio, the green emission of ZnO nanosheets increases significantly while the NBE emission is weakened. In addition, the UV peaks were blue-shifted with the decrease of P_2_O_5_ weight ratio. Similar to the effect of oxygen flow rate, it would be due to the crystal quality improvement of ZnO changing the band tailing effect, which is observed from the change of FWHM of XRD peaks with P doping. The NBE exciton UV emission and defect-related deep-level green emission compete with each other. The improvement of the crystal quality (reduction in the impurities and structural defects such as oxygen vacancies and dislocations etc.) can enhance the near-band-edge emission with reduction or vanishing of the green emission [33]. Similar phenomena that a high-doping level increasing the UV emission meanwhile decreasing the green emission were observed in Al- and P-doped ZnO by other groups in both films and nanostructures [32, 34].

### Growth Mechanism of ZnO Nanosheets with Branched Structures

**Fig. 7:**
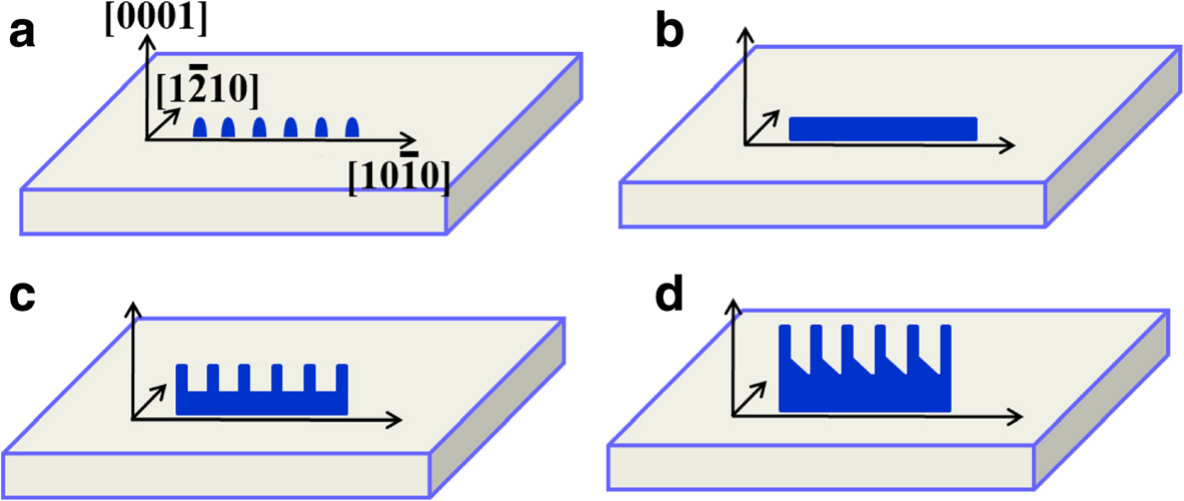
Schematic diagram of growth process of a ZnO nanosheet branched structure, **a** formation of Zn nuclei, **b** P doping induces the growth of ZnO nanoribbons, **c** oxygen promotes the growth of nanoteeth, and **d** planar filling of ZnO nanosheets

The growth of ZnO nanosheets goes through three stages: P doping altering the growth direction of ZnO and forming nanoribbons, oxygen facilitating the growth of nanoteeth, and planar filling widening the backbone of ZnO nanosheets. Figure 7 shows the schematic diagram of the growth process of ZnO nanosheets. The reactions in the growth system are:

Reaction area:

$$ \begin{array}{l}\mathrm{Z}\mathrm{n}\mathrm{O}\left(\mathrm{s}\right)+\mathrm{C}\left(\mathrm{s}\right)\to \mathrm{Z}\mathrm{n}\left(\mathrm{g}\right)+\mathrm{C}\mathrm{O}\left(\mathrm{g}\right)\hfill \\ {}{\mathrm{P}}_2{\mathrm{O}}_5\left(\mathrm{s}\right)+\mathrm{C}\left(\mathrm{s}\right)\to \mathrm{P}\left(\mathrm{g}\right)+\mathrm{C}\mathrm{O}\left(\mathrm{g}\right)\hfill \\ {}\mathrm{Z}\mathrm{n}\mathrm{O}\left(\mathrm{s}\right)+\mathrm{C}\mathrm{O}\left(\mathrm{g}\right)\to \mathrm{Z}\mathrm{n}\left(\mathrm{g}\right)+{\mathrm{CO}}_2\left(\mathrm{g}\right)\hfill \end{array} $$

Substrate area:

$$ \mathrm{Z}\mathrm{n}\left(\mathrm{g}\right)+{\mathrm{O}}_2\left(\mathrm{g}\right)+\mathrm{P}\left(\mathrm{g}\right)\to \mathrm{Z}\mathrm{n}\mathrm{O}:\mathrm{P}\left(\mathrm{s}\right) $$

The $\left\{0001\right\},\left\{11\overline{2}0\right\},\kern0.5em \mathrm{and}\kern0.5em \left\{10\overline{1}0\right\}$ planes are frequently reported surface planes of wurtzite ZnO nanostructures, and the corresponding fast growth directions are $\left[0001\right],\kern0.24em \left[11\overline{2}0\right],\;\mathrm{and}\ \left[10\overline{1}0\right],$, respectively. The surface energy of the three directions follows the order of $\mathrm{E}\left\{0001\right\}>\mathrm{E}\left\{11\overline{2}0\right\}>\mathrm{E}\left\{10\overline{1}0\right\}$ [23]. At the initial stage, Zn vapor is reduced by graphite and carried to sapphire substrates to form Zn nuclei, tending to accumulate along the [0001] direction as shown in Fig. 6a. Meanwhile, P atoms as dopants incorporate in ZnO to partially occupy the O sites. However, the Zn-P bond length (2.18 Å) is significantly larger than that of ZnO bond (1.93 Å) [12]; thus, P atoms introduce lattice strain when occupying the substitutional O sites. Strain relaxation occurs along the perpendicular direction of [0001] direction, which alters the preferential growth direction of ZnO from $\left[0001\right]\ \mathrm{t}\mathrm{o}\;\left[10\overline{1}0\right]$ lattice direction. As a result, ZnO nanoribbons along the $\left[10\overline{1}0\right]$ direction are grown, as shown in Fig. 7b. The side (top and bottom) surfaces of nanoribbons are terminated with Zn and O, respectively, forming a positively charged Zn-terminated (0001) plane and negatively charged O-terminated $\left(000\overline{1}\right)$ plane [35].

The formation of a branched structure is attributed to the diffusion process in a supersaturated environment, in which oxygen facilitates the growth of nanoteeth along the [0001] direction to form a ZnO nanosheet branched structure. The oxygen flow gas can react with Zn vapor to form ZnO gas molecules effectively resulting in a large supersaturation degree, which activates secondary growth sites and heterogeneous nucleation on the Zn-terminated side surface via providing proper sites for Zn clustering or local enrichment of Zn to self-catalyze the growth of nanoteeth along the [0001] direction [17]. When more Zn atoms are absorbed on the (0001) surface, they could accumulate from more new Zn clusters, accelerating the growth of nanoteeth. Conversely, the O-terminated $\left(000\overline{1}\right)$ plane at the other edge of the nanoribbons without self-catalyzed growth cannot form nanoteeth. Consequently, ZnO nanosheets with a ribbon-like backbone and one-side-parallel nanoteeth are fabricated, as shown in Fig. 7c.

With further growth of ZnO nanosheets, P_2_O_5_ will also be present because of its high vapor pressure; thus, sufficient P atoms continue to promote the growth of nanoribbons, and the intervals between nanoteeth is filled continuously due to the preferential condensation of vapors at concave corners between nanoteeth. Finally, the planar filling widens the ribbons of ZnO nanosheets, as shown in Fig. 7d. It should be pointed out that Au plays an important role in the synthesis of ZnO nanostructures. Zero-dimensional Au nanoparticles catalyze the growth of vertical ZnO nanowires. In other words, without P inducing lattice strain or oxygen promotion effect, ZnO normally would grow along the [0001] direction to form uniform nanowires, as shown in Fig. 1e.

## Conclusions

In this work, we demonstrated the controllable synthesis of ultrathin ZnO nanosheets by a simple CVD method. P doping and O_2_ reaction gas are found to be essential for the growth of ZnO nanosheets. P doping induces lattice distortion in ZnO by substituting oxygen sites, and the strain relaxation alters the growth direction of ZnO from the $\left[0001\right]\ \mathrm{t}\mathrm{o}\ \left[10\overline{1}0\right]$ direction. Oxygen can facilitate the growth of ZnO nanosheets along the lateral directions. The ZnO nanosheets with a large surface area are found to have outstanding performance for various applications such as UV detecting, photocatalytic reactions, gas sensing, etc.
